# Incentive motivation improves numerosity discrimination: Insights from pupillometry combined with drift-diffusion modelling

**DOI:** 10.1038/s41598-020-59415-3

**Published:** 2020-02-13

**Authors:** Annika Dix, Shu-Chen Li

**Affiliations:** 1 Faculty of Psychology, Chair of Lifespan Developmental Neuroscience, Technische Universität Dresden, 01062 Dresden, Germany; 2 Centre for Tactile Internet with Human-in-the-Loop (CeTI), Technische Universität Dresden, 01062 Dresden, Germany

**Keywords:** Perception, Reward

## Abstract

Recent studies show that training the approximate number system (ANS) holds promise for improving symbolic math abilities. Extending this line of research, the present study aims to shed light on incentive motivation of numerosity discrimination and the underlying mechanisms. Thirty-two young adults performed a novel incentivized dot comparison task, that we developed, to discern the larger of two numerosities. An EZ-diffusion model was applied to decompose motivational effects on component processes of perceptual decision-making. Furthermore, phasic pupil dilation served as an indicator of the involvement of the salience network. The results of improved accuracy and a higher information accumulation rate under the reward condition suggest that incentive motivation boosts the precision of the ANS. These novel findings extend earlier evidence on reward-related enhancements of perceptual discrimination to the domain of numerosity perception. In light of the Adaptive Gain Theory, we interpret the results in terms of two processes of gain modulation driven by the locus coeruleus-norepinephrine system. Specifically, the reward-induced increase in pupil dilation may reflect incentive modulation of (i) salience attention during reward anticipation towards incentivized stimuli to upregulate stimulus processing that results in a larger drift rate; and (ii) response caution that leads to an increased decision threshold.

## Introduction

“Number sense” refers to the intuitive ability to process numerical information without consciously dealing with symbolic representations of numbers^1^. This sense of numerosity has been widely studied and discussed as a foundational perceptual function that serves higher-order cognitive processes for the acquisition of abstract numerical concepts and mathematical skills in humans^2^. A subcomponent of this function is the approximate number system (ANS), which allows for a quick, albeit inexact, estimation of the number of sensory (e.g., visual or auditory) objects. The precision of ANS correlates with mathematic achievements throughout the lifespan^3,4^ and has been investigated in earlier training studies that were aimed at enhancing individuals’ ANS precision and related symbolic math abilities^5^. Such training usually only relies on accuracy-based feedback^6–8^, although reward incentive has been validated as a potent modifier of performance in many other cognitive and sensory domains (for reviews, see^9–11^). So far, systematic research on incentive motivation of the precision of the ANS has not been undertaken. Thus, the aim of the current study is twofold: (i) investigating whether incentive motivation improves numerosity discrimination; and (ii) understanding mechanisms underlying the potential incentive benefits.

Incentive motivational regulation implicates neural activities in the brain’s reward network, which broadly comprises various cortical and striatal regions that are richly innervated by the mesolimbic and nigrostriatal dopamine (DA) projections (for reviews, see^12–16^). According to the Incentive Salience hypothesis^13^, reward-related DA signals in the mesolimbic system are assumed to strengthen the perceptual salience assigned to the neural representations of reward-associated objects; thereby, incentive motivation directs attention towards incentivized, though not necessarily task-relevant, stimuli (e.g., task-irrelevant features associated with reward during visual search)^17^. In a different vein, reward-induced perceptual enhancement has also been considered to stem from the impact of endogenous, top-down attentional regulation, which biases perception. The detection or discrimination of anticipated reward cues is facilitated through such processes^10,18–21^. In line with these ideas, the present study aimed to examine the potential benefit of reward-related incentive salience on enhancing the precision of numerosity representations and thereby improving perceptual discrimination. In so doing, earlier studies on incentive-driven attention in visual search can be extended to the domain of numerosity perception. To better capture mechanisms underlying incentive motivation, other than behavioural performance we also assessed task-related pupil dilation (PD) – a measure that has been linked to activities in the brain’s salience network during reward processing^22,23^.

In earlier research, pupil size has primarily been used as a proxy to indicate norepinephrinergic activities subserving affective (e.g., arousal) and cognitive (e.g., attention or mental load) functions (see^24,25^ for reviews). According to the Adaptive Gain Theory (AGT) of locus coeruleus-norepinephrine (LC-NE) function by Aston-Jones and Cohen^26^, task-evoked (phasic) PD reflects neural activity in the LC that favours the processing of task-relevant information over task-irrelevant explorative behaviour in the environment. The AGT proposes that the LC receives direct inputs about costs and feedback in the current task from cortical structures, such as the anterior cingulate cortex (ACC) as part of the reward system. In line with this postulate, a study by Schneider *et al*.^23^, which combined pupillometry with functional magnetic resonance imaging (fMRI), found increased activation in the dorsal ACC to be positively correlated with PD during the expectation of monetary rewards. Another study by Manohar and Husain^27^ further lends support for the roles of the reward network and mesolimbic DA in mediating the association between incentive motivation and reward-induced PD. Using a speeded saccade task with monetary incentives, it was shown that pupillary reward sensitivity in patients with Parkinson’s disease was restored after being treated with a DA agonist. In the present study, we were particularly interested in whether phasic PD evoked by a reward cue may reflect effects of incentive modulation during a numerosity discrimination task. Furthermore, we examined whether incentive benefits on performance would be related to reward-induced phasic increase in pupil size.

One typical paradigm for studying numerosity discrimination is the non-symbolic dot comparison task, in which two arrays of dots are presented on each trial and the participants have to decide which array contains more dots^3,4^. In the research on perceptual decision-making, the drift-diffusion model (DDM) has been used widely to dissociate the decision process into distinct components^28^. In recent years, its application in the field of numerosity perception has gained increased attention. Park and Starns^29^ demonstrated that one parameter of this model, the drift rate (*v*), could serve as a measure of the precision of the ANS. It represents the efficiency of sensory evidence accumulation about the to-be-compared quantities. Since the DDM allows the decomposition of the decision process into subcomponents, it has advantages not only for studying numerical processing at the behavioural level with more specificity, but also for examining the mechanisms underlying reward-related modulations of the ANS. For instance, the drift rate of perceptual decision depends on various stimulus features, such as difficulty or perceptual salience. Further, it can also be affected by the person’s attentional focus^28^. Consistent with the Incentive Salience hypothesis, Spaniol *et al*.^30^ observed greater drift rates (i.e., more efficient information accumulation) in the reward condition as compared to a neutral condition of a perceptual discrimination task.

Besides the drift rate, DDMs have another parameter that reflects the decision criterion (threshold) at which point the necessary amount of evidence is reached for a given decision to be taken. This decision threshold, also known as the boundary separation (*a*) parameter, can be adapted to optimize accuracy per unit of time based on feedback favouring response speed over the accuracy or vice versa^31^ (cf. optimal-decision theories^32^). In a new reinforcement learning-based DDM, Fontanesi *et al*.^33^ recently observed a lower decision threshold for highly attractive options in a value-based decision-making task. This is in accordance with the findings by Cavanagh *et al*.^34^, who showed a reduced decision threshold in the most appetitive condition of a probabilistic selection task. Interestingly, in this latter study, which combined a hierarchical Bayesian DDM with pupillometry, larger PD was associated with increased response caution (i.e., a higher decision threshold) in high-conflict situations (i.e., appetitive conflict in the most appetitive and aversive conflict in the most aversive condition, respectively). Furthermore, the non-decision time (*t*_*ER*_) parameter of DDMs captures response time (RT) that is not directly related to the decision process (e.g., sensory encoding or response execution)^35^. Due to its broad definition, this parameter theoretically reflects all non-decision related processes, which makes it – in terms of the meaning – less specific. It has been associated with various psychological constructs including attention and may thus be subject to reward-related modulation as well.

**Figure 1:**
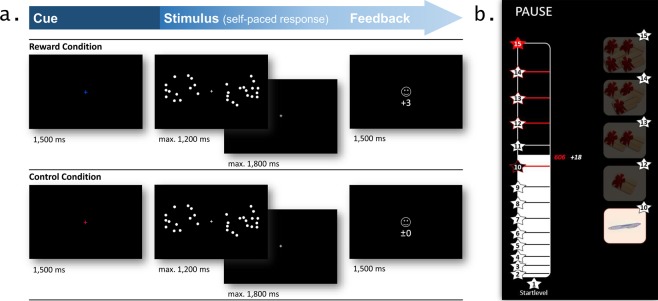
Schematic representation of the experimental task: **(a)** Trial scheme of the incentivized dot comparison task depicting the three phases (i.e., incentive cue, stimulus, feedback) of a correctly answered trial for both the reward (top) and the control (bottom) condition; **(b)** Scoring scheme shown after each block giving an overview about the points the participant collected in reward trials during the last block (white number) and the accumulated total points (red number and white bar) as well as the obtained bonus winning level (levels 1–15) and the corresponding items (pencil or vouchers; right panel) the participant had already won (see Methods section for more details about the task).

Taken together, the twofold purpose of the present study was to investigate the impact of incentive motivation on numerosity discrimination and to examine the underlying mechanisms by combining pupillometric measures with the application of a simplified version of the DDM, the EZ-diffusion model^36^. To this end, we developed a new incentivized dot comparison task (see Fig. 1 and the Methods section for more details). We expected that reward incentive would improve task performance by increasing the salience of the representations of different numerosities^13^. Relatedly, we assumed task-evoked PD to be larger during reward anticipation and when processing stimuli in rewarded trials compared to unrewarded trials. Moreover, we expected that a stronger reward modulation effect at the psychophysiological level would be associated with reward-induced performance outcomes (i.e., higher levels of bonus won in the task)^23^. Other than effects related to reward anticipation, we hypothesized that larger PD would reflect increased discrimination difficulty, that is, smaller differences between the quantities of the to-be-compared dot arrays in our paradigm (e.g., a ratio between the two arrays of 10:9 vs. 5:4).

Regarding the subcomponents of the decision process, we hypothesized that reward would lead to an increase in drift rate (*v*)^30^, indicating enhanced precision of the ANS^29^ as well as affecting the decision threshold (boundary separation *a*) and the non-decision time (*t*_*ER*_)^35^. To date, findings on the effects of reward on boundary separation are still somewhat mixed and studies on non-decision time are still underrepresented. Thus, it is harder to make clear predictions about these two parameters. In contrast, evidence of the effects of task difficulty or cognitive demand is more unequivocal. For instance, in conditions with higher processing conflicts that are subsequently cognitively more demanding, information accumulation should be slowed and the individuals probably would raise their response caution, while responses may generally also take more time (e.g., longer non-decision time)^28^. We thus anticipated drift rate (*v*) to decrease and boundary separation (*a*) and non-decision time (*t*_*ER*_) to increase with greater task difficulty.

As for potential interactions between the effects of incentive and task difficulty on performance or pupillary response, conceptually we expected the difficulty effect to be attenuated under the reward condition if the effect of reward is in sharpening the representation of numerosity. Empirical evidence on this interaction in other domains of functioning is still mixed. In a study on speech processing, for instance, performance improvement under reward was only observed in the more demanding tasks. Further, in these tasks, PD was reduced for high but not low reward levels^37^. The authors interpret these findings in terms of strategic effort control to achieve a rewarding goal. By contrast, in the perceptual domain, a fMRI study investigating the influence of reward on visual discrimination showed the opposite pattern, with reward yielding a larger performance enhancement in easier tasks than in the most difficult one^38^.

## Results

### Behavioural performance

**Figure 2:**
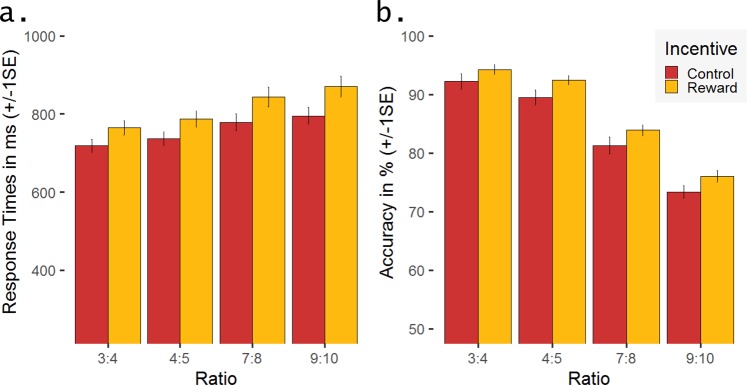
Behavioural performance: Mean and standard error (SE) for **(a)** response times (RT) in milliseconds and **(b)** accuracy in per cent, both separated for the two incentive conditions (reward vs. control) and the four ratio conditions.

Analysing RT data using a linear mixed-effects model revealed main effects of Incentive, *F*(1,217) = 117.00, *p* < 0.0001, η_p_² = 0.35, and Ratio, *F*(3,217) = 59.64, *p* < 0.0001, η_p_² = 0.45. Reward and more difficult numerosity discrimination (smaller differences between the two dot arrays) slowed participants’ decision time. Pairwise t-tests showed no significant difference between the two easiest (ratio 4:3 vs. 5:4; *p* = 0.32, one-tailed) and the two hardest (ratio 8:7 vs. 10:9; *p* = 0.32, one-tailed) ratio conditions, respectively (all other *p*s < 0.04, one-tailed; see Fig. 2a). The linear mixed-effects model for accuracy also revealed main effects of Incentive, *F*(1,217) = 23.44, *p* < 0.0001, η_p_² = 0.10, and Ratio, *F*(3,217) = 251.02, *p* < 0.0001, η_p_² = 0.78. Participants’ answers were more often correct in rewarded trials than in the control condition as well as in trials with larger differences between the two dot arrays, i.e., the easier discriminations (all *p*s < 0.02, one-tailed; see Fig. 2b). The Incentive × Ratio interaction effect was not significant, neither for RTs (*p* = 0.22) nor for accuracy (*p* = 0.94).

### Parameters of the drift diffusion model

**Figure 3:**
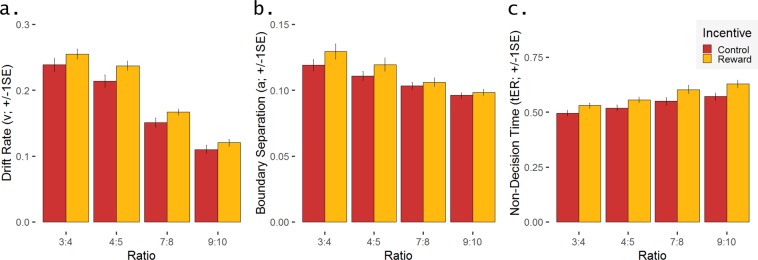
Model parameters of the decision-making process: Mean and standard error (SE) for **(a)** drift rate (*v*), **(b)**. boundary separation (*a*), and **(c)** non-decision time (*t*_*ER*_), separately for the two incentive conditions (reward vs. control) and the four ratio conditions.

Analysing the drift rate (*v*) of the EZ-DDM using a linear mixed-effects model revealed main effects of Incentive, *F*(1,217) = 19.30, *p* < 0.0001, η_p_² = 0.08, and Ratio, *F*(3,217) = 262.33, *p* < 0.0001, η_p_² = 0.89. The information accumulation rate was higher in trials with reward compared to the control trials as well as in trials with a larger difference between the two dot arrays (all *p*s < 0.003, one-tailed; see Fig. 3a). The linear mixed-effects model for boundary separation (*a*) also revealed main effects of Incentive, *F*(1,217) = 14.10, *p* = 0.0002, η_p_² = 0.06, and Ratio, *F*(3,217) = 54.57, *p* < 0.0001, η_p_² = 0.43. The value of the boundary separation parameter was larger – reflecting a more conservative response criterion – in trials with reward compared to the control trials as well as in trials with larger differences between the two dot arrays (all *p*s < 0.03, one-tailed; see Fig. 3b). This latter finding seems to contradict the frequently reported positive correlation between the decision threshold and RTs, which, by contrast, were larger in trials with smaller differences between the two dot arrays. However, computing Pearson’s product-moment correlations between RTs and boundary separation (*a*) separately for each condition yielded the expected positive correlations (with 0.57 ≤ *r* ≤ 0.79, all *p*s < 0.01; Holm-correction for multiple testing). The linear mixed-effects model for non-decision time (*t*_*ER*_) revealed main effects of Incentive, *F*(1,217) = 70.56, *p* < 0.0001, η_p_² = 0.25, and Ratio, *F*(3,217) = 51.37, *p* < 0.0001, η_p_² = 0.41. The value of *t*_*ER*_ was larger (indicating slower non-decision time) in trials with reward as well as in trials with smaller differences between the two dot arrays. Pairwise t-tests showed no significant difference between the two easiest (ratio 4:3 vs. 5:4; *p* = 0.12, one-tailed) and the two hardest (ratio 8:7 vs. 10:9; *p* = 0.12, one-tailed) conditions (with all other *p*s < 0.03, one-tailed; see Fig. 3c). For all three parameters of the DDM, no significant Incentive × Ratio interaction effect was observed (*p* = 0.68 for *v*, *p* = 0.17 for *a*, and *p* = 0.36 for *t*_*ER*_).

### Pupillometry measures

**Figure 4:**
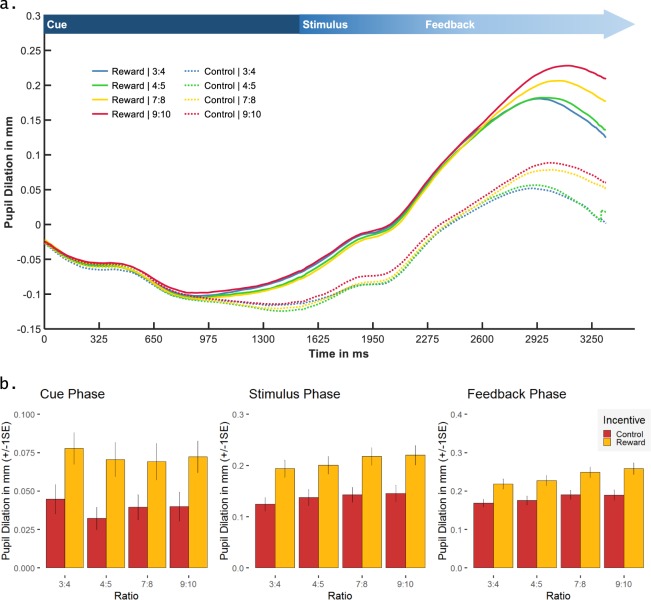
Pupillometry measures: **(a)** Mean pupil dilation (in millimetres) across the time course of the trial (in milliseconds), stimulus-locked to the onset of the cue (zero point in time), and separately for the different conditions (incentive condition: reward vs. control; four ratio conditions); arrow at the top indicates the associated phase of the trial; **(b)** mean and standard error (SE) for the pupil dilation (in millimetres) measured at the peak of the pupil response during reward anticipation (cue phase; left), numerosity discrimination (stimulus phase; middle), and feedback processing (feedback phase; right), separately for the two incentive conditions and the four ratio conditions.

The mean courses of the pupil response over a trial for the different conditions are shown in Fig. 4a. Linear mixed-effects models for peak PD in the three trial phases revealed main effects of Incentive during reward anticipation (cue phase), *F*(1,217) = 96.37, *p* < 0.0001, η_p_² = 0.31, numerosity discrimination (stimulus phase), *F*(1,217) = 192.09, *p* < 0.0001, η_p_² = 0.47, and feedback processing (feedback phase), *F*(1,217) = 150.98, *p* < 0.0001, η_p_² = 0.41. The PD was larger for trials with reward compared to the control condition in all three phases of the task, reflecting incentive modulation effects during reward anticipation, stimulus, and feedback processing. The main effects of Ratio were only observed during numerosity discrimination, *F*(3,217) = 4.58, *p* = 0.004, η_p_² = 0.06, and feedback processing, *F*(3,217) = 9.67, *p* < 0.0001, η_p_² = 0.12 (*p* = 0.22 during reward anticipation). Pairwise t-tests showed no significant difference between the four ratio conditions, neither during the stimulus (all *p*s > 0.51, one-tailed) nor during the feedback phase (*p*s > 0.08, one-tailed). Nevertheless, polynomial contrasts testing the linear component of the main effect Ratio (using the aov function of the stats package in R^39^) revealed increased peak PD during feedback processing the smaller the difference between the two dot arrays, *F*(1,252) = 6.33, *p* = 0.01, η_p_² = 0.02 (see Fig. 4b). The polynomial contrast for the linear component of the Ratio effect during stimulus processing did not reach significance (*p* = 0.13). Further, no significant interaction between Incentive and Ratio was observed, neither for peak PD during reward anticipation (*p* = 0.84) nor during numerosity discrimination (*p* = 0.83) or feedback processing (*p* = 0.45).

The analyses of the correlation between incentive modulation of the peak PD and the level of winning bonus obtained in the task showed a positive relationship between the two. Greater increases in PD during reward anticipation was associated with a higher level of win at the end of the experiment (ρ = 0.39, *p* = 0.04, one-tailed). This relation was not observed during the phase of numerosity discrimination (*p* = 0.77, one-tailed) or feedback processing (*p* = 0.77, one-tailed). For further details regarding descriptive statistics of all dependent variables, see Table S1 in Supplementary Results.

## Discussion

Math achievements depend on non-symbolic numerical abilities^3,4^ and can be improved by training the precision of the ANS^5^. While early theories of numerosity discrimination suggest reward as a potent modifier of numerosity perception^40^, to the best of our knowledge, the present study is the first to systematically investigate whether incentive motivation can enhance the precision of the ANS during a novel incentivized dot comparison task designed by us. We expected incentive salience to facilitate numerical representations and thus improve numerosity discrimination^13^. To scrutinize mechanisms underlying effects of incentive modulation, we recorded phasic PD, a measure known to be associated with the activity of the brain’s salience network^23^. Furthermore, we fit the EZ-diffusion model^36^ to the behavioural data to separate the decision process into subcomponents. The main findings of this study are that reward enhanced numerosity discrimination as indicated by higher accuracy and more efficient information accumulation (i.e., higher drift rate). Moreover, reward slowed the discrimination process, which was accompanied by raised response caution as reflected in an increased decision threshold (i.e., greater boundary separation). The duration of non-decision time, partly reflecting sensorimotor processes, was also extended by reward. Of note, the pupillary response was modulated by reward as well: the PD was larger in rewarded compared to non-rewarded trials, both during numerosity discrimination and feedback processing. Moreover, the increase of PD was already observed during the phase of reward anticipation when the reward cue was presented. This reward-evoked PD correlated positively with the level of bonus wins that the participants obtained at the end of the experiment. We discuss these main findings in more detail below.

This study shows that incentive motivation manipulated by monetary rewards enhances non-symbolic numerical discrimination as assessed with our incentivized dot comparison task. This finding replicates and extends earlier evidence on reward-related enhancements of perceptual and cognitive processing^10^ to the domain of numerosity perception. The better numerosity discrimination under reward (i.e., higher accuracy) suggests that incentive motivation facilitates perceptual representations of the to-be-compared quantities and thereby increases the precision of the ANS. At the same time, it needs to be kept in mind that slowed decision speed – as observed in the present study – could also be indicative of an effect of speed-accuracy trade-off. By fitting the data to an EZ-diffusion model that takes into account both performance accuracy and RTs, we were able to shed light on the effects of incentive motivation on different components of the decision process. The drift rate of the model constitutes a measure of the precision of numerosity representation^29^. We observed greater drift rates in rewarded compared to unrewarded trials, which supports the interpretation of more distinctive numerical representations due to incentive motivation. Reward-related modulations of the drift rate during perceptual discrimination have been observed before by Spaniol *et al*.^30^. They compared the effects of incentives on perceptual efficiency in younger and older adults during colour perception. Likewise, the attentional DDM by Krajbich and Rangel^41^ illustrates that the value assigned to an object seems to be reflected in the drift rate of their model, which was increased in favour of attended objects. Following the Incentive Salience hypothesis^13^, in the present study we assumed that the reward cue would increase attention during rewarded trials and thus enhance the salience of the numerical representations. Results of the pupillary data are well in line with this notion of incentive salience being an underlying mechanism of reward-evoked enhancement of numerosity perception.

In particular, first, during the reward anticipation phase we observed increased PD during rewarded relative to the control trials. This may reflect enhanced resource allocation due to incentive motivation. In a recent combined pupillometry/fMRI study, PD associated with the anticipation of monetary rewards turned out to be coupled with neuronal activity in the salience network of the brain, particularly in the dorsal ACC^23^. The ACC plays a crucial role in performance monitoring (for a review, see^42^). According to the AGT by Aston-Jones and Cohen^26^, the ACC regulates the allocation of attentional resources and has direct projections to the LC to optimise the level of task-engagement and related outcome. Further, together with other regions of the salience network, the ACC has been widely discussed as being a key player in the bottom-up detection of salient information, given its effects in triggering attentional processes and motor preparation (for a review, see^43^). Besides, the consistency of our findings with the Incentive Salience hypothesis supports previous evidence indicating that pupil response measures can capture the interplay between the dopaminergic reward system and the LC-NE system^27,44^. Interestingly, the extent to which PD was enhanced by the presence of reward cues correlated with the level of bonus won by the participants at the end of the experiment. Consequently, the interplay between the dopamine reward and the LC-NE attention systems may play an important role in promoting numerosity perception for optimal goal-directed behaviour^26^. Admittedly, the effect observed in the current study was weak and our interpretation needs to be verified in future studies that examine the relationship between the pupillary and behavioural effects in more detail and their underlying neural mechanisms directly. For instance, the correlations between PD data and diffusion model parameters will need to be investigated in larger samples. Multi-modal approaches that integrate positron-emission tomography (PET) receptor imaging with fMRI or electroencephalography (EEG) measures as well as methods for modelling neurophysiological data as a diffusion process (for a review, see^45^) would need to be considered.

Second, other than effects during reward anticipation, we showed that incentive motivation also elevated PD during the phases of numerosity discrimination and feedback processing. Moreover, besides the effects of reward, we observed a ratio-dependent modulation of PD, with PD being larger when the difference of the two to-be-discriminated dot arrays was smaller. This effect is in line with findings from previous studies showing impacts of feedback and cognitive conflict on PD (e.g.,^34,46^). Of note, given the established roles of the ACC, this brain region might assess performance-related information like internal conflict during stimulus processing or external feedback (e.g., anticipated or received reward)^42^ to regulate LC activation for task engagements (reflected in changes of PD; cf. the AGT^26^) in the current context of numerosity discrimination. As to be expected from previous studies (cf.^29^), the ratio between the to-be-compared quantities also affected performance: smaller differences (higher conflicts) between the dot arrays resulted in longer RTs and lower accuracy. One study by Cavanagh *et al*.^34^ linked conflict-related PD with adjustments of decision threshold in terms of higher response caution in high-conflict situations. This could also explain the ratio-dependent RT slowing that was accompanied by increased PD in the present study. Nevertheless, we did not find an effect of ratio on increasing response caution. Thus, other explanations of this finding may still be necessary.

An earlier study^47^ fitted RT data by standard and moderately adapted diffusion models in conditions of high response or reinforcement conflict and showed that the data can be best accounted for when boundary separation (i.e., the decision threshold) and non-decision time increase with response or reward conflict. In agreement with these findings, conflict in the present study (i.e., smaller differences between the quantities in the dot arrays) was associated with slowed non-decision time. However, as stated above boundary separation decreased with increasing conflict. This seems to contradict the increase in RTs observed here and the frequently reported positive correlation between decision threshold and RTs. Apart from the seemingly reversed impacts of task difficulty (i.e., ratio) on both measures in our study, individual differences in RTs and decision thresholds correlated positively in our data as to be expected. The observed effect of task difficulty in decreasing boundary separation of the decision process resembles the reward-rate optimal boundary values proposed in another study^48^. This study compared younger and older adults’ ability to trade off speed and accuracy to optimize performance in a numerosity task. The calculated reward-rate optimal boundary values, representing an objective criterion defining the decision threshold for an optimal speed-accuracy trade-off, decreased with increasing difficulty. Although participants typically show wider decision thresholds than this objective criterion, it is known that accuracy feedback and extensive task practice can help to bring one’s decision threshold closer to these values. Given the effects reported in the earlier study^48^, the design of our task (performance feedback after every trial, 768 trials in total) might explain the atypical result pattern that we observed and reflect a close to optimal decision threshold.

Furthermore, the EZ-diffusion model revealed increases in boundary separation and non-decision time during rewarded compared to unrewarded trials and ensuing decreases in response speed. Raising the decision threshold in response to reward has also been linked with mesolimbic DA functioning. For instance, a study with attention deficit hyperactivity disorder (ADHD) patients showed increased response caution in a reinforcement learning task after medication that modulates DA-levels^49^. As in the present study, non-decision time became longer in ADHD patients after DA medication^49^. It should be noted that our findings appear to contradict recent evidence by Fontanesi *et al*.^33^, who reported lower decision thresholds for objects with high values. Our results are also inconsistent with insights from a modelling approach by Manohar *et al*.^50^, who demonstrated reward-related reductions of precision costs in fast decisions. Notwithstanding, the current results can be integrated well into the framework of optimal-decision theories^32^. Participants in our study were rewarded for correct decisions, where decision speed only played a minor part due to the relatively long durations of stimulus presentation and the response time window. It is likely that accuracy was favoured over RT in our numerosity discrimination task for several other reasons. Participants received accuracy-related feedback at the end of each trial and incorrect decisions were “punished” (bonus points lost). Further, unlike the highly valuable situation in the study by Fontanesi *et al*.^33^, information in our task was characterized by high uncertainty. Taken together, incentive motivation in our task increased the rate of perceptual evidence accumulation (reflected in drift rate), raised response caution (reflected in boundary separation), and lengthened non-decision time. We interpret these findings by referring to two processes that could underlie the reward-related modulation of the ANS precision: (i) the LC-NE driven gain modulation of stimulus processing due to incentive salience that is reflected in the increased drift rate; and (ii) a strategic process via top-down control processes of the prefrontal cortex to increase response caution as reflected in the increased boundary separation. In light of previous findings, both processes might be triggered by the ACC. These interpretations, however, need to be further validated in future studies.

In conclusion, combining pupillometry with drift-diffusion modelling provides insights into mechanisms underlying incentive modulation of numerosity perception. We showed that incentive motivation enhances the precision of ANS in young adults performing an incentivized dot comparison task. This extends earlier evidence on the reward-related enhancement of perceptual discrimination^10^ to the processing of numerical information. These findings are in line with the Incentive Salience hypothesis^13^ and the AGT by Aston-Jones and Cohen^26^. In future studies, it might be worthwhile to develop an experimental paradigm that also systematically manipulates the valence of the outcome. It has been shown that the threat of losses increases arousal compared to the promise of gains (e.g.,^51^), which should be reflected in a more pronounced pupil response. Manipulating the valence of the outcome would provide additional information about incentive-related modulations of the precision of the ANS dependent on the type of reward. Furthermore, the consideration of reward feedback effects could yield complementary information, as research in other domains indicates outcome effects on performance that might even surpass reward anticipation effects (cf.^52^). The EZ-diffusion model, which we combined with the pupillometry measures in the present study, considered the central parameters of the DDM. These parameters were also assumed to play crucial roles in modelling perceptual efficiency (i.e., drift rate) and performance optimization (i.e., boundary separation) under incentive motivation. Future studies might still want to apply a full DDM or modified versions thereof to explore further aspects of the perceptual decision process during numerosity discrimination. Such aspects of interest may be attention in an attentional DDM^41^ or modulatory effects on the decision threshold by allowing the parameter of the boundary separation to collapse over time^47^. A full DDM can also account for drift rate variability, which is not considered by the EZ-diffusion model applied here and may have resulted in an underestimation of the drift rate^36^ in the present study (see Supplementary Methods). Moreover, it might be worthwhile to examine whether adjustments of the decision threshold still occur in a task that emphasizes processing speed. If this adjustment is also one mechanism underlying the performance modulatory effects of incentive motivation, it should be present even when the allowed response time interval is limited. Besides, an independent effect of reward on the drift rate in a task design with a response deadline would lend further support to the notion that the enhanced precision of ANS is due to incentive salience of the numerical representations. These open questions notwithstanding, the present study identified incentive motivation as a promising modifier of numerosity discrimination, with potential implications for mathematical education. To put these lab-based results into educational practice, further studies on the impact of incentive motivation are necessary. They would need to take age-related changes in the ANS^3^, brain maturation and their interplay into account. Here, the role of developmental trajectories of the dopaminergic system during childhood and adolescence^53^ might be of particular interest.

## Methods

### Participants

Thirty-three students at Technische Universität (TU) Dresden aged 18–33 years took part in this study. Due to severe signal loss during pupil tracking in one participant, 32 participants were included in the analysis (17 females; age: *M* = 23.75 years, *SD* = 4.68). An a priori power calculation indicated sufficient power with a reasonably low probability of a type II error and a high likelihood to detect potential effects of reward in a sample of this size (required sample size of *N* = 16). The power calculation was based on prior knowledge of the following: (i) the impact of reward on the performance in an earlier version of the incentivized non-symbolic dot comparison task that we validated in a pilot study; and (ii) reported changes in peak PD in response to reward for relevant cognitive functions (e.g., decision-making, number processing) in young adults^54–57^. Only one study specified the effect size of such a change^54^, which was smaller than the effects observed in our behavioural pilot study. Thus, the power calculation using G*Power 3.1^58^ was based on this one effect size of f = 0.70 with two measurements (reward vs. control), a zero correlation between these two (*r* = 0.00, which is the most demanding case), a significance level of α = 0.05, and the statistical power 1-β = 0.95.

All participants had normal or corrected-to-normal vision, no history of neurological or psychiatric diseases, and were not taking any medication. The study was approved by the ethics committee of the TU Dresden (EK 55022017) under the Declaration of Helsinki and performed following the relevant guidelines and regulations. All participants gave informed written consent before the investigation. After testing, they received 15 Euro for their participation plus the bonus they had earned during the incentivized non-symbolic dot comparison task (prizes with a value of 9.12 Euro on average).

### Experimental paradigm

We developed an incentivized non-symbolic dot comparison task (see Fig. 1a), in which numerosities of two quantities in different ratios were presented. Reward was manipulated to explore the effects of incentive motivation on the precision of the number sense. In this task, we instructed the participants to decide which of two arrays has more dots by pressing the left or the right control key on a keyboard. Each trial started with an incentive cue – a pink or blue fixation cross (luminance: 1.17 cd/m²) – displayed in the centre of a computer screen for 1,500 ms, followed by the presentation of the two arrays of dots flanking a grey fixation cross (luminance: *M* = 0.97 cd/m²). Participants were asked to make their decision as quickly and accurately as possible. The dot arrays were presented for 1,200 ms and the participants’ responses were allowed until 3,000 ms after stimulus onset. The response was followed by emoticon-based accuracy feedback (smiley vs. sad emoticon; luminance: 1.17 cd/m²) for a period of 1,500 ms. Besides, numbers indicating points earned (or lost) on that trial were shown below the feedback symbol. The two incentive conditions (reward vs. control) were signalled by the colour of the cue at the beginning of each trial. In trials of the reward condition, each correct or erroneous response resulted in gaining or losing, respectively, three points. In trials of the control condition, correct or erroneous responses did not result in points gained or lost, thus the value below the feedback symbol was zero. The allocation of the colour of the cue to one of the two conditions was counterbalanced across participants. Trial types (reward vs. control) were randomized within a block. Each block consisted of 24 trials and the task included 32 blocks, with a short 10-second break between blocks. In the first 5 seconds of each break, the thus far accumulated points from reward trials were shown along with the current bonus level obtained and the distance to the next level (see Fig. 1b). In total, there were 15 levels; higher levels were associated with a higher bonus level, which correspond to one of five possible win options at the end of the experiment: a pen (1) or a voucher for a local shopping mall worth 10 Euro (2), 15 Euro (3), 20 Euro (4), or 25 Euro (5). All instructions of the task were presented in white letters on a black background. The dot arrays and the feedback at the end of each trial were displayed in grey on a black background as well. The maximum field area of the dot arrays encompassed 7.5° visual angles in diameter. The number of dots in each array varied between 12 and 32, with a ratio between the two arrays of 4:3 (3:4), 5:4 (4:5), 8:7 (7:8) or 10:9 (9:10). For further details on stimulus generation and procedures of experiment controls, see Supplementary Methods. The task took about 50 min to complete. RTs, error rates and PD during the task were recorded as dependent variables (see Supplementary Methods for more details on data acquisition).

### Data analyses

The behavioural data (RTs, accuracy) were analysed with the statistical software packages SPSS Statistics for Windows, Version 25.0 (IBM Corp., Armonk, NY, USA) and R (Version 3.4.3)^39^ in R Studio 1.1.414 (RStudio, Inc.). First, an outlier analysis was conducted: correct trials for which RTs were 3.5 standard deviations (SDs) above or below the individuals’ mean, as well as items for which the RTs were 3.5 SDs above or below the sample mean of the respective condition (reward vs. control for the different ratios), were removed. Using these criteria only 0.89% of the trials were eliminated. By and large, condition did not affect the number of outliers, except for significantly more outliers in the ratio condition of 10:9 compared to 8:7 in rewarded trials or in the ratio condition of 10:9 in unrewarded trials. Afterwards, the responses were averaged for each condition and participant. To further characterize the perceptual decision process during numerosity discrimination, we applied the EZ-diffusion model^36^ to our data separately for each condition and participant. It estimates three parameters, i.e., drift rate (*v*), boundary separation (*a*) and non-decision time (*t*_*ER*_), based on the individual’s decision accuracy as well as the mean and variance of RTs of the correct responses. Following recommendations by Ratcliff and McKoon^28^, trials with RTs under 250 ms or above 1,500 ms were excluded. Thus, another 1.66% of the trials were eliminated for the model-based analysis. The suitability of the data for the application of the EZ-diffusion model was checked^36^, which indicated that the drift rate might be underestimated in all conditions but one (i.e., unrewarded trials with a ratio of 4:3 between the two arrays). Since this concerned most conditions, the comparisons of effects across conditions would not be affected. Nevertheless, we considered this point when interpreting the results (see also Supplementary Methods for more details).

For the processing of the pupillary data, Matlab 9.3, R2017B (The MathWorks, Inc., MA, USA), SPSS 25 and R packages in R Studio 1.1.414 were used. Before the statistical data analysis, pupillary data (average pupil diameter across the left and right eye) was cleaned following standard procedures^59,60^: trials with excessive blinking were discarded. There were no systematic differences in the distribution of pupillary artefacts across the experimental conditions. Small blinks were replaced by cubical interpolations. After discarding outliers and artefacts, 72.96% of all trials remained for the separate analyses of the three different phases of trials (reward anticipation, numerosity discrimination, feedback). For each condition and participant, we computed a stimulus-locked pupillary response for the reward anticipation and the feedback phase (duration: 1500 ms after cue or feedback presentation) and a response-locked pupillary response for the numerosity discrimination phase (duration: 1500 ms until 200 ms after button press). Pupillary responses were smoothed by an unweighted 5-point moving-average filter and baseline corrected for each trial subtracting the average pupil diameter of a 200 ms period before phase onset. On average, pupillary responses were calculated from 70 trials per condition per participant with a minimum of 10, which was assumed to constitute a reliable measurement with sufficiently reduced noise for the statistical analyses on the peak PD of each phase. Due to the missing inter-trial-interval and the ongoing recovery of the pupil at the beginning of a trial, baseline correction resulted in negative values during the cue phase. Therefore, the dilation relative to the minimum of the pupil diameter was chosen to define the peak in this phase (cf. Fig. 4a).

Linear mixed-effects models with maximum-likelihood estimation and participants as random intercepts were conducted using the lme function from the nlme package in R^61^. These models considered within-subject effects of the factors Incentive (i.e., reward vs. control condition) and Ratio (i.e., the four different ratios between the dot arrays) on (i) performance measures (RTs, accuracy), (ii) the parameter estimates of the EZ-diffusion model (*v*, *a*, *t*_*ER*_) and (iii) the peak PD separately for the three different trial phases (cue, stimulus, feedback). Post-hoc multiple comparisons were carried out using pairwise t-tests (Holm-correction for multiple testing^62^). Effect sizes are reported using partial eta squared (η_p_²) following the approach and recommendations by Fern and Monroe^63^ and Maxwell *et al*.^64^. In case the Saphiro-Wilk-test or the visual inspection using Q-Q-plots revealed that the models’ residuals were not normally distributed, permutation tests using the lmer function from the lme4 package^65^ and permanova from the predictmeans package in R^66^ were performed. Since the analyses showed comparable results for the permutated models, we only report results from analyses using the linear mixed-effects models. We also analysed correlations between incentive modulation of pupil response and a behavioural measure of the overall performance on the rewarded trials. For this, we calculated Spearman’s rank correlation coefficient for the reward-related peak PD modulation in the three different trial phases and the bonus winning level at the end of the experiment (achieved win option 1 to 5; Holm-correction for multiple testing). The reward-related peak PD modulation was defined as the relative change in peak PD according to the formula $({peak}\,{P}{{D}}_{{reward}}-{peak}\,{P}{{D}}_{{control}})/[({peak}\,{P}{{D}}_{{reward}}+{peak}\,{P}{{D}}_{{control}})/2]$. For all analyses, a significance criterion of *p* ≤ 0.05 (two-tailed) was chosen (one-tailed only for tests with clear directional hypotheses based on findings from prior studies). The datasets generated and analysed in the present study (i.e., anonymous behavioural and pupillometric data) will be made available for research purposes by the corresponding author upon request.

## Supplementary information

Supplementary Material.
