# Supplementary material for: Incentive motivation improves numerosity discrimination: Insights from pupillometry combined with drift-diffusion modelling
Source: Sci Rep. 2020 Feb 13;10:2608. doi: 10.1038/s41598-020-59415-3 (PMC7018719; doi:10.1038/s41598-020-59415-3)
Supplement: Supplementary file 1 — Supplementary Material. [file 41598_2020_59415_MOESM1_ESM.pdf]

Incentive motivation improves numerosity discrimination: Insights from pupillometry combined with drift-diffusion modelling

Annika Dix* and Shu-Chen Li

Supplementary Results

Descriptive statistics are displayed in Table S1, including means and standard deviations of the behavioural performance (RTs, accuracy), the model parameters of the decision-making process (drift rate, boundary separation, and non-decision time) as well as the peak PD in the three trial phases (cue phase, stimulus phase, feedback phase) separated for the two incentive conditions (reward vs. control) and the four ratio conditions.

Table S1. Descriptive statistics: mean (*M*) and standard deviation (*SD*) for the behavioural performance measures, the model parameters of the decision-making process, and the pupillometry measures.

	**Reward**				**Control**			
**Ratio**	**3:4**	**4:5**	**7:8**	**9:10**	**3:4**	**4:5**	**7:8**	**9:10**
RTs								
M (ms)	764.49	787.24	843.85	870.41	718.44	736.54	778.65	795.04
SD (ms)	106.48	114.16	139.44	149.49	96.31	101.74	120.64	121.01
Accuracy								
M (%)	94.28	92.46	83.90	76.04	92.21	89.44	81.30	73.39
SD (%)	4.85	4.61	5.12	5.47	7.41	7.30	8.06	5.90
Drift rate (v)								
M	0.25	0.24	0.17	0.12	0.24	0.21	0.15	0.11
SD	0.04	0.04	0.03	0.03	0.06	0.05	0.04	0.03
Boundary separation (a)								

M	0.13	0.12	0.11	0.10	0.12	0.11	0.10	0.10
SD	0.03	0.03	0.02	0.01	0.03	0.02	0.02	0.01
Non-decision time (t_{ER})								
M	0.53	0.56	0.60	0.63	0.50	0.52	0.55	0.57
SD	0.07	0.08	0.10	0.10	0.07	0.07	0.09	0.10
Peak pupil dilation – cue phase								
M (mm)	0.08	0.07	0.07	0.07	0.04	0.03	0.04	0.04
SD (mm)	0.06	0.06	0.07	0.06	0.05	0.04	0.05	0.05
Peak pupil dilation – stimulus phase								
M (mm)	0.19	0.20	0.22	0.22	0.12	0.14	0.14	0.15
SD (mm)	0.09	0.10	0.10	0.11	0.07	0.09	0.08	0.09
Peak pupil dilation – feedback phase								
M (mm)	0.22	0.23	0.25	0.26	0.17	0.18	0.19	0.19
SD (mm)	0.08	0.08	0.08	0.08	0.06	0.07	0.07	0.07

Supplementary Methods

Experimental Paradigm

The number of dots and the ratio between the arrays were matched in an orthogonal design, the position (left vs. right) of the larger dot array was randomly assigned and each stimulus was presented twice in the course of the experiment, once in the reward condition and once in the control condition. Additionally, we controlled two other dimensions – size and spacing – of the dot arrays which were manipulated orthogonally to the number following the approach by DeWind et al.[1]. For one-half of the trials, the size of the dots but not the total surface area was matched between the dot arrays. For the other half of the trials, the total surface area of the two dot arrays was the same, whereas dot sizes differed. Independently from that, in half of the trials, the sparsity of the dots but not the diameter and resulting total field area was equated for the dot arrays, whereas for the other half of the trials it was the

other way around. Thereby, participants were encouraged to make their decisions on the dimension of number.

Data Acquisition

Participants were tested separately in a quiet moderately illuminated room (background luminance ca. 350 lx). To control for pupil-influencing factors (e.g., drug consumption, medication, psychiatric and neurological dysfunction; cf. Loewenfeld, 1993), participants completed a corresponding paper-and-pencil questionnaire (simultaneous background luminance adaptation). Then, they were seated in front of a computer screen (size of the display: 23, display resolution: 1680 × 1050) at a distance of 65 cm. The testing was conducted on a computer using the software Presentation 18.1 (Neurobehavioral Systems Inc, Albany, CA) running on a Microsoft® Windows® 7 operating system. This computer recorded the behavioural data and was connected to a second computer using the software Tobii Studio 3.4.5 (Tobii Technology AB, Stockholm, Sweden), which was controlled by the Tobii Workspace Extension for Presentation to record the measurement of the pupil diameter. The pupil diameter was tracked by an infrared binocular eye-tracking system, a Tobii Pro TX300 (Tobii Technology AB, Stockholm, Sweden) using dark pupil tracking with a sampling frequency of 300 Hz to provide an estimate of the pupil diameter of the left and right eye in millimetres. The eye-tracking unit was attached to the lower edge of the computer screen, where this integrated screen setup allows participants to move the head naturally in front of it and does not require them to be positioned in a chinrest. As we also recorded gaze data (accuracy: 0.4° of visual angle) and participants' eye blink rate, each individual's gaze on the eye-movement monitor was first calibrated using a 9-point calibration and afterwards recorded during five minutes, in which the participant fixated a grey fixation cross presented on a black background (1.17 cd/m²) to assess their spontaneous eye blink rate [2]. Before instructing participants on the incentivized non-symbolic dot comparison task, which is in the focus of the present work, a psychometric assessment was conducted with paper-and-pencil

tests to measure participants' math anxiety (Abbreviated Math Anxiety Scale, AMAS[3]), motivational systems for behavioural inhibition and activation (Behavioural Inhibition/ Behavioural Activation System Scales, BIS/BAS[4]), need for cognition (Need for Cognition Scale, NFC[5]), performance on a symbolic numerical discrimination test[6] and a mathematic achievement test (Calculation Subtest of the Woodcock-Johnson-Revised Tests of Achievement, WJ-R[7]). A second experimental task, a non-symbolic arithmetic task adopted from Hyde et al.[8] as well as Park and Brannon [9], and further psychometric computer-based measures on perception speed (Identical-Pictures Test[10]), verbal intelligence (Mehrfach-Wahl-Wortschatz-Test B, MWT-B[11]) and spatial working memory[12] followed at the end of the in a total 3-hour session. Finally, participants were compensated for their participation and received the prize they won in the incentivized dot comparison task.

Data Analyses

Inspection of the key characteristics of the RT distributions (mean, variance, kurtosis and skewness) indicated ex-Gaussian distributions throughout the data and accordingly, their suitability for the application of the EZ-diffusion model [13]. Further, a comparison of the relative speed of correct and error responses for the different target positions of the larger dot array by computing ANOVAs with Target (left vs. right) and Response (correct vs. error) on RTs in the different conditions for each participant did not point to the prioritization of one answer button. This is in line with the assumption of the EZ-diffusion model of an unbiased starting point. However, in 91 out of 256 cases, Mann-Whitney-U test revealed that RTs were significantly faster for correct compared to error responses (without correction for family-wise error), which is an indicator of across-trial variability in drift rate that is not considered by the EZ-diffusion model. Wagenmakers et al.[13] could show that the EZ-diffusion model underestimates all parameters, particularly the drift rate, in such cases. In the present study, faster correct than error responses occurred in all conditions except for unrewarded trials with a ratio of 4:3 between the two arrays. This was indicated by another eight Mann-Whitney-U

tests that we performed across the whole sample computing one test for each condition without separating between participants. Therefore, the resulting underestimation of the parameters should be taken into account for their interpretation.

References

1 DeWind, N. K., Adams, G. K., Platt, M. L. & Brannon, E. M. Modeling the approximate number system to quantify the contribution of visual stimulus features. *Cognition* **142**, 247-265, doi:10.1016/j.cognition.2015.05.016 (2015).

2 Doughty, M. J. Assessment of short-term variability in human spontaneous blink rate during video observation with or without head / chin support. *Clin Exp Optom* **99**, 135-141, doi:10.1111/cxo.12326 (2016).

3 Hopko, D. R., Mahadevan, R., Bare, R. L. & Hunt, M. K. The Abbreviated Math Anxiety Scale (AMAS): Construction, Validity, and Reliability. *Assessment* **10**, 178-182, doi:10.1177/1073191103010002008 (2003).

4 Carver, C. S. & White, T. L. Behavioral inhibition, behavioral activation, and affective responses to impending reward and punishment: The BIS/BAS scales. *Journal of Personality and Social Psychology* **67**, 319-333, doi:10.1037/0022-3514.67.2.319 (1994).

5 Bless, H., Fellhauer, R. F., Bohner, G. & Schwarz, N. in *ZUMA-Arbeitsbericht 1991/06* (ed Methoden und Analysen -ZUMA- Zentrum für Umfragen) (1991).

6 Nosworthy, N., Bugden, S., Archibald, L., Evans, B. & Ansari, D. A two-minute paper-and-pencil test of symbolic and nonsymbolic numerical magnitude processing explains variability in primary school children's arithmetic competence. *PloS one* **8**, e67918, doi:10.1371/journal.pone.0067918 (2013).

7 Woodcock, R. W. & Johnson, M. B. *Woodcock-Johnson Psycho-Educational Battery–Revised*. (Riverside Publishing, 1989).

8 Hyde, D. C., Khanum, S. & Spelke, E. S. Brief non-symbolic, approximate number practice enhances subsequent exact symbolic arithmetic in children. *Cognition* **131**, 92-107, doi:10.1016/j.cognition.2013.12.007 (2014).

9 Park, J. & Brannon, E. M. Training the approximate number system improves math proficiency. *Psychol Sci* **24**, 2013-2019, doi:10.1177/0956797613482944 (2013).

10 Ekstrom, R. B., French, J. W., Harman, H. & Derman, D. *Kit of factor-referenced cognitive tests (rev. ed.)*. (Educational Testing Service, 1976).

11 Lehrl, S. *MWT-B: Mehrfach-Wahl-Wortschatz-Test B*. (Straube, 1977).

12 Nagel, I. E. *et al.* Performance level modulates adult age differences in brain activation during spatial working memory. *Proceedings of the National Academy of Sciences of the United States of America* **106**, 22552-22557, doi:10.1073/pnas.0908238106 (2009).

13 Wagenmakers, E., Van Der Maas, H. L. J. & Grasman, R. P. P. P. An EZ-diffusion model for response time and accuracy. *Psychon Bull Rev* **14**, 3-22, doi:10.3758/BF03194023 (2007).
